# Construction and application of home dietary management program for postoperative patients with preventive ileostomy for rectal cancer

**DOI:** 10.3389/fnut.2025.1639987

**Published:** 2025-09-12

**Authors:** Jianhua Zhu, Yuanjuan Shen, Juyun Li, Sainan Wang, Wenjuan Shang, Min Sun

**Affiliations:** Affiliated Hospital of Nanjing University of Chinese Medicine (Jiangsu Province Hospital of Chinese Medicine), Nanjing, Jiangsu Province, China

**Keywords:** ileostomy, rectal cancer, dietary management, malnutrition, high-output stoma

## Abstract

**Background:**

Rectal cancer is a highly prevalent malignant tumor, and surgery is one of the main treatment methods. Although preventive ileostomy can reduce postoperative complications, it is also prone to cause malnutrition and other problems.

**Objective:**

To construct and validate an early postoperative home dietary management program for patients with preventive ileostomy for rectal cancer, aiming to improve their nutritional status and mitigate clinical ileostomy complications.

**Methods:**

An evidence-based dietary management program was developed, covering dietary transition, nutrient intake, and fluid management. A non-concurrent controlled study was conducted with 66 patients at the Affiliated Hospital of Nanjing University of Chinese Medicine. The intervention group received personalized one-on-one dietary guidance (including pre-discharge assessment of dietary habits, individualized meal planning, and weekly WeChat-based follow-up for food diary reviews) in addition to routine health education, while the control group received routine education only.

**Results:**

At 1 month postoperatively, the intervention group showed significantly better outcomes than the control group in serum albumin (41.00 g/L vs. 38.00 g/L, *p* = 0.010, *r* = 0.368), lymphocyte count (1.30 × 10?/L vs. 1.10 × 10?/L, *p* = 0.009, *r* = 0.374), and PG-SGA scores (9.85 vs. 10.94, *p* = 0.037, *Cohen's d* = 0.525). The intervention group had lower incidences of high-output stoma (HOS; 6.06% vs. 18.18%, OR = 0.29, 95% *CI* = 0.05–1.56, *p* = 0.258) and peristomal moisture-associated skin damage (PMASD; 18.18% vs. 24.24%, OR = 0.69, 95% *CI* = 0.21–2.29, *p* = 0.547), with positive clinical trends despite no statistical significance.

**Conclusion:**

This home dietary management program can effectively improve short-term postoperative nutritional status in patients, and also plays a positive role in reducing the occurrence of HOS and PMASD.

## 1 Background

Colorectal cancer ranks third in global incidence and second in mortality among malignant tumors. In China, its incidence ranks second and mortality fourth, accounting for 28.2% of global cases (1, 2). Surgical treatment is a main approach for rectal cancer, and with the continuous development of surgical techniques, sphincter-preserving surgery for rectal cancer has been widely adopted. Preventive ileostomy, used in such surgeries, serves to decrease the incidence of postoperative anastomotic dehiscence and attenuate the severity of pelvic sepsis in the event of fistula formation (3). However, ileostomy presents significant challenges. In the absence of the colon's ability to absorb water and electrolytes, a substantial volume of digestive fluids is expelled through the ileostomy. This physiological alteration renders patients vulnerable to high-output stoma (HOS), malnutrition, metabolic imbalances, and immune dysregulation. Studies have found that 23.7% of patients develop HOS after ileostomy (4), while the incidence of malnutrition is as high as 79.09% (5).

To cope with these issues, some ileostomy patients adjust their dietary intake by avoiding certain foods or reducing food intake to reduce stoma output or avoid issues like odor. Additionally, the lack of professional home rehabilitation dietary guidance leads to further problems. For instance, 39.7% of patients with peristomal moisture-associated skin damage (PMASD) within 1 month after surgery still consumed semi-liquid diets (6), which is significantly associated with increased stoma output. This, in turn, leads to insufficient energy intake or nutritional imbalance, thereby increasing malnutrition risk (7). Thus, professional dietary guidance interventions are needed to reduce stoma output, alter stool characteristics, and improve patients' nutritional status.

Although existing guidelines provide dietary management recommendations for ileostomy patients (8, 9), their content is fragmented and lacks systematic dietary advice. This hinders the rapid and effective clinical implementation of dietary management, and patients may even receive inconsistent dietary advice. Therefore, this study aims to construct and evaluate an evidence-based early postoperative home rehabilitation dietary management program for patients with preventive ileostomy after rectal cancer surgery, providing a reference for dietary management interventions.

## 2 Methods

### 2.1 Construction of early postoperative dietary management program for patients with preventive ileostomy for rectal cancer

#### 2.1.1 Establishing the research team

The research team consisted of 8 members: 1 head nurse from gastrointestinal oncology surgery responsible for project guidance, 2 specialist physicians responsible for disease diagnosis and treatment, 1 nutritionist responsible for reviewing and modifying the dietary management program, 3 stoma therapists responsible for patient education and follow-up management, and 1 research nurse primarily responsible for overall project coordination, consultation, and data collection. The research team held regular meetings to advance the project with close collaboration.

#### 2.1.2 Construction of the early postoperative dietary management program

This study summarized evidence on dietary management interventions for patients after preventive ileostomy for rectal cancer through evidence-based methods, extracted relevant content, and formed a preliminary draft of the dietary management program. Ten experts were selected to complete the consultation, resulting in the program. Eight patients were selected for a pilot test. The program was adjusted based on pilot results, ultimately forming the final dietary management program (Table 1).

**Table 1:** Dietary management program for patients with preventive ileostomy.

**Item**	**Content**
Diet type selection and recording	1. Within 1 week after discharge, patients can consume semi-liquid diets and gradually transition to regular diets, following principles of low residue, low fat, and low fiber. Avoid spicy, irritating, and fried foods, maintain nutritional balance and dietary diversity to establish dietary tolerance and navigate the dietary transition period. 2. Use tools such as 24-h dietary recall forms, food diaries, or food frequency questionnaires to record patients' dietary intake types, quantities, and frequencies.
Nutrient selection	3. Energy supply: determine energy supply based on patient needs. If HOS occurs, the patient's energy target should be increased by at least 30–50% above normal intake to compensate for intestinal losses. 4. Energy composition: 40–50% from carbohydrates, 20% from protein, and 30–40% from fat. 5. Consume starchy foods such as rice and noodles; limit high-monosaccharide foods like candy, honey, jam, jelly, and high-sugar beverages. 6. Increase protein intake: protein requirement is 1.5–2.5 g/(kg·d). 7. Use medium- and short-chain fatty acids instead of long-chain fatty acids, ensuring adequate essential fatty acids and fat-soluble vitamins. 8. Limit dietary fiber intake. Recommend soluble fiber foods such as viscous, non-fermentable, gel-forming soluble fiber supplements (e.g., psyllium husk). Reduce insoluble fiber intake from high-fiber foods (e.g., celery, asparagus), indigestible fiber peels (e.g., apples), corn, raw cabbage, dried fruits, nuts, popcorn, casing meats, mushrooms, coconut, etc., to avoid obstruction. 9. Recommend oral multivitamin and mineral supplements, such as vitamin B12, iron, calcium, liquid magnesium, zinc, manganese, and selenium.
Fluid management	10. Oral fluid intake: 1,500–1,700 mL. If HOS occurs, increase daily intake by 500–750 mL above the average recommended intake for the general population. 11. Types of oral fluids: limit hypotonic fluids (e.g., water, tea, coffee, alcohol) and hypertonic fluids (e.g., fruit juice, cola) to < 1,000 mL per day. If HOS occurs, oral rehydration salts can be used.
Dietary behaviors	12. Eat small, frequent meals to maintain appetite. Divide meals into 5–6 times, scheduled during the daytime to reduce nocturnal stoma output and ensure nighttime rest. 13. Avoid eating too quickly during meals; take small bites, chew thoroughly, and avoid drinking water during meals.
HOS assessment and monitoring	14. Stool assessment: assess and record daily stoma excretion characteristics and volume. If stoma output >1,500 mL/day, consider HOS. 15. Urine assessment: record daily urine volume and color, ensuring urine output >1,000 mL/day. 16. Dehydration assessment: monitor for signs and symptoms of dehydration (e.g., dry mouth, decreased urine output, dark concentrated urine, dizziness when standing, significant fatigue, abdominal cramps). Seek medical attention if symptoms occur. 17. Weight monitoring: weigh at least once weekly.

### 2.2 Application of the dietary management program

#### 2.2.1 Study design and participants

A non-concurrent controlled study was conducted in the general surgery and gastrointestinal oncology surgery wards of Nanjing University of Chinese Medicine Affiliated Hospital. Patients undergoing preventive ileostomy for rectal cancer between July 2024 and February 2025 were included. Thirty-three patients from July-November 2024 were assigned to the control group, and thirty-three patients from December 2024-February 2025 were assigned to the intervention group. Inclusion criteria: (a) Diagnosed with rectal cancer, underwent radical tumor resection with preventive ileostomy, expected survival >1 year; (b) Age 18–75 years; (c) Conscious with unimpaired communication. Exclusion criteria: (a) Eating disorders; (b) Severe primary heart, liver, lung, kidney, blood diseases or other serious conditions affecting survival; (c) Participation in other similar clinical trials. The study was approved by the Ethics Committee of Nanjing University of Chinese Medicine Affiliated Hospital (2024NL-181-02). Sample size calculation based on comparing means of two groups: n_1_ = n__2__ = 2[(μα + μβ)σ/δ]^2^, with α = 0.05 (two-sided), β = 0.10 (one-sided). From preliminary results, δ = 3.40, σ = 3.82, yielding n_1_ = n__2__ ≈27. Considering 20% attrition, the adjusted sample size was 33 per group (total 66).

#### 2.2.2 Intervention methods

##### 2.2.2.1 Control group

Received routine health education. Before discharge, they received home care manuals and were educated by stoma therapists about ileostomy characteristics and dietary points. They were informed about stoma clinics and instructed to follow up regularly. Telephone follow-ups were conducted within 1 week after discharge.

##### 2.2.2.2 Intervention group

In addition to the control group's intervention, they received in-depth communication with stoma therapists and nutritionists 2–3 days before discharge to understand preoperative eating habits and care needs. They received personalized one-on-one dietary guidance tailored to their eating habits and preventive ileostomy characteristics. On discharge day, stoma therapists reviewed the education and provided additional guidance as needed. Patients received a home dietary management manual and joined a stoma patient WeChat group for nutrition and dietary questions. Patients uploaded weekly food diaries and bowel movement records for monitoring.

#### 2.2.3 Outcome measures

Patient demographics and tumor-related indicators (age, gender, Body Mass Index (BMI), education level, tumor stage) were recorded. Outcome measures included primary and secondary endpoints assessed pre-intervention and 1 month post-discharge.

##### 2.2.3.1 Primary outcomes

Nutritional indicators (serum albumin, lymphocyte count, PG-SGA, BMI). Fasting blood samples were collected in the morning and analyzed at the hospital's clinical laboratory to determine serum albumin levels and lymphocyte counts. PG-SGA were assessed by the nutritionist. BMI was calculated using the formula: BMI = Weight (kg)/Height (m)^2^. The nutritionist measured the patients' height and fasting weight using the Meilen hospital-specific integrated ultrasonic height and weight measuring instrument (Model: MSG003).

##### 2.2.3.2 Secondary outcomes

Incidence of HOS and PMASD within 1 month postoperatively. HOS was defined as stoma output >1,500 mL/24 h.

#### 2.2.4 Statistical analysis

SPSS 25.0 was used. Normally distributed continuous data were expressed as mean±SD and analyzed with independent *t*-tests; non-normally distributed data were expressed as median (P25, P75) and analyzed with Mann-Whitney U tests. Categorical data were described as counts and percentages, analyzed with χ^2^ tests, Fisher's exact test, or rank-sum tests. *p* < 0.05 was considered statistically significant.

## 3 Results

### 3.1 Comparison of general patient characteristics

A total of 66 patients were included in this study, all of whom completed the 1-month postoperative follow-up. There were no statistically significant differences in the general characteristics between the two groups (*p* > 0.05, Table 2).

**Table 2:** Comparison of general characteristics.

**Variable**	**Intervention group (*n* = 33)**	**Control group (*n* = 33)**	**Statistic**	***p-value***
**Gender**, ***n*** **(%)**			*χ2* = 0.363	0.547
Female	6 (18.2)	8 (24.2)		
Male	27 (81.8)	25 (75.8)		
Age (years), mean ± SD	61.88 ± 8.89	60.03 ± 11.52	*t* = 0.73	0.468
**Marital status**, ***n*** **(%)**			-	0.492^a^
Single	1 (3.13)	0 (0.0)		
Married	31 (93.94)	33 (100.0)		
Widowed	1 (3.13)	0 (0.0)		
**Hypertension**, ***n*** **(%)**			*χ2* = 0.067	0.796
No	22 (66.7)	21 (63.6)		
Yes	11 (33.3)	12 (36.4)		
**Diabetes**, ***n*** **(%)**			*χ2* = 3.264	0.071
No	29 (87.9)	23 (69.7)		
Yes	4 (12.1)	10 (30.3)		
**Educational level**, ***n*** **(%)**			*Z* = −0.724	0.469
Primary school or below	7 (21.2)	5 (15.15)		
Junior high school	14 (42.4)	15 (45.45)		
Senior high school	7 (21.2)	4 (12.12)		
Associate's degree	4 (12.1)	8 (24.24)		
Bachelor's degree	1 (3.0)	1 (3.03)		
**Tumor stage**, ***n*** **(%)**			*Z* = −0.274	0.784
Stage I	2 (6.1)	2 (6.1)		
Stage II	9 (27.3)	7 (21.2)		
Stage III	20 (60.6)	23 (69.7)		
Stage IV	2 (6.1)	1 (3.0)		

^a^Fisher's exact test.

### 3.2 Comparison of nutritional indicators

Comparison results of nutritional indicators between the two groups at discharge and 1 month postoperatively are shown in Table 3. There were no significant differences in nutritional indicators between the two groups at baseline. At 1 month postoperatively, significant differences were observed in serum albumin, lymphocyte count, and PG-SGA scores (*P* < 0.05), but no significant difference was found in BMI changes between the two groups.

**Table 3:** Comparison of nutritional status.

**Indicator**	**Intervention group (*n* = 33)**	**Control group (*n* = 33)**	**Statistic**	***p-value***	***Effect size***
**Serum albumin (g/L), M (P25, P75)**					
At discharg	38.00 (35.1, 41.9)	37.30 (35.5, 40.3)	*Z* = −0.783	0.434	*r* = 0.112
At 1 month	41.00 (39.0, 42.5)	38.00 (36.5, 41.3)	*Z* = −2.572	0.010	*r* = 0.368
**Lymphocyte Count (10** ^9^ **/L), M(P25, P75)**					
At discharge	1.20 (0.9, 1.4)	1.08 (0.8, 1.4)	*Z* = −0.629	0.530	*r* = 0.09
At 1 month	1.30 (1.0, 1.8)	1.10 (0.8, 1.3)	*Z* = −2.611	0.009	*r* = 0.374
**BMI (kg/m**^2^**), mean** ±**SD**					
At discharge	23.79 ± 3.40	24.91 ± 3.05	*t* = −1.395	0.168	cohen's d = 0.404
At 1 month	22.45 ± 3.17	23.66 ± 2.81	*t* = −1.628	0.109	cohen's d = 0.346
**PG-SGA, mean** ±**SD**					
At discharge	11.97 ± 2.07	12.06 ± 2.81	*t* = −0.150	0.881	cohen's d = 0.037
At 1 month	9.85 ± 1.33	10.94 ± 2.62	*t* = −2.133	0.037	cohen's d = 0.525

### 3.3 Comparison of complication rates

Comparison results of HOS and PMASD incidence rates within 1 month postoperatively between the two groups are shown in Table 4. Two patients in the control group were readmitted due to dehydration caused by HOS. Although the incidence of HOS and PMASD was higher in the control group than in the intervention group, there was no statistically significant difference between the two groups.

**Table 4:** Incidence of HOS and PMASD (**%)**.

**Complication**	**Intervention group (*n* = 33)**	**Control group (*n* = 33)**	***Statistic***	***OR***	***95% CI***	***p-value***
HOS, *n* (%)	2 (6.1)	6 (18.2)	-	0.29	0.05–1.56	0.258^a^
PMASD, *n* (%)	6 (18.2)	8 (24.2)	*χ2* = 0.363	0.69	0.21–2.29	0.547

a:Fisher's exact test.

## 4 Discussion

### 4.1 Scientific rationale and practical value of the home dietary management program

The development of this home dietary management program was grounded in current evidence-based guidelines and clinical best practices. It integrates key aspects such as staged dietary transition, macronutrient and micronutrient optimization, fluid and electrolyte balance, fiber modulation, eating behavior coaching, and stoma output monitoring. This comprehensive and structured approach addresses the complex nutritional needs of ileostomy patients and provides actionable strategies for home implementation.

In clinical settings, patients with ileostomy often report confusion and anxiety regarding dietary choices. Prior surveys indicate that approximately 55% of patients feel overwhelmed by postoperative dietary restrictions and uncertain about appropriate food selection (10). Although guidelines suggest resumption of low-residue semi-liquid diets within the first week and regular diets by the second postoperative week (11), study findings reveal that 16.74% of patients remain on liquid or semi-liquid diets beyond 2 weeks postoperatively, predisposing them to malnutrition (12). This discrepancy highlights a gap between guidelines and patient behaviors, underscoring the need for individualized, behavior-oriented interventions.

This program addresses that gap by offering tailored, structured guidance. Through collaborative efforts between stoma therapists and clinical dietitians, the program respects preoperative eating habits while promoting gradual adaptation through weekly follow-ups. The emphasis on patient autonomy and continuous engagement helps establish self-management capabilities. These findings are aligned with previous studies (13), yet this study adds value by operationalizing dietary guidance into a home-based model and confirming its feasibility and acceptability among patients post-ileostomy.

### 4.2 Program effectively supports early nutritional recovery

Both groups demonstrated significant improvements in serum albumin, total lymphocyte count, and PG-SGA scores 1 month after surgery, with the intervention group showing more pronounced gains. This reinforces the nutritional benefit of structured home dietary interventions. The observed outcomes are consistent with Lin's findings (14), yet our study expands on them by incorporating micronutrient and fiber management into the intervention, which was less emphasized in prior work.

Interestingly, BMI declined slightly in both groups at 1 month, mirroring the findings of Vasilopoulos et al. (15), who attributed weight loss to postoperative catabolism and fluid shifts. Kim et al. (16) reported continued BMI reduction up to 40 days post-surgery, hypothesizing that intestinal adaptation post-ileostomy plays a role. These findings suggest that while short-term nutritional biomarkers may recover with intervention, anthropometric parameters like BMI require longer follow-up and possibly adjusted energy targets.

The high stoma output characteristic of ileostomy leads to nutrient losses—particularly protein, electrolytes, and micronutrients—contributing to protein-energy malnutrition in over half of patients (17). This program addressed this by recommending high-protein intake (1.5–2.5 g/kg/day), with energy intake increased by 30–50% based on individual needs. Compared to earlier studies (18, 19), this study introduced a more nuanced approach by incorporating stoma output monitoring and adjusting macronutrients in real-time, showing favorable trends in immune and nutritional markers.

Moreover, the program's emphasis on refined starches and low-fiber foods for output control was supported by previous research (18, 19). However, unlike Lee's case-specific report (19), our study systematically applied this strategy across a broader cohort, providing stronger evidence for generalizability. Additionally, the inclusion of soluble fiber, known to enhance nutrient retention and absorption (20), added a novel dimension to traditional dietary recommendations for ileostomy patients.

This study also incorporated medium- and short-chain fatty acids (MCFAs/SCFAs) as alternatives to long-chain fats, based on bile acid metabolism disturbances observed in ileostomy patients (21). These changes are not commonly emphasized in existing nutritional protocols. SCFAs' role in promoting mucosal immunity and maintaining barrier integrity further underscores the long-term benefits of this dietary strategy. Deficiencies in micronutrients such as B12, iron, and zinc, which affect up to 31% of patients (22), were proactively addressed through supplementation—highlighting the program's comprehensiveness and preventive orientation.

### 4.3 Positive trend in preventing complications: toward clinical relevance

While the reduction in high-output stoma (HOS) incidence in the intervention group (6.06% vs. 18.18%) was not statistically significant (*p* = 0.258), the observed trend suggests a clinically meaningful reduction in risk. Notably, two patients in the control group required rehospitalization due to severe HOS, underscoring the real-world relevance of dietary management in complication prevention. This finding is particularly important as most previous studies have not quantitatively assessed HOS incidence in relation to dietary interventions.

The intervention emphasized behaviorally informed strategies—frequent small meals, thorough chewing, and avoidance of hypotonic fluids—to modulate intestinal transit and stoma output. While earlier studies (18) have identified these factors individually, the present program integrates them into a patient-centered home regimen, allowing for early detection of dehydration through daily monitoring and prompt intervention. Compared to generalized hydration advice, our program adapted the WHO oral rehydration model to suit ileostomy patients, yielding significant improvements in urea and creatinine levels (23). This practical approach offers a replicable template for clinical guidance and extends the application of standardized hydration strategies to surgical patients.

Regarding peristomal moisture-associated skin damage (PMASD), while no statistically significant differences were found (18.18% vs. 24.24%, *p* = 0.547), the lower incidence in the intervention group supports the potential of nutritional and behavioral measures in reducing skin complications. As highlighted by Indrebø et al. (24), PMASD is multifactorial, influenced by stoma care practices, nutrition, comorbidities, and skin integrity. Our findings suggest that integrating dietary management into a comprehensive stoma care protocol may contribute to better skin outcomes, though further research is needed to confirm these associations.

## 5 Limitations

This study used a non-concurrent controlled design, which may be subject to time bias or uncontrollable confounding factors. The lack of statistically significant differences in complication rates between groups is mainly attributed to the small sample size and low statistical power. Additionally, the follow-up period was only 1 month postoperatively, insufficient to evaluate the program's long-term effects and potential impact on ultimate tumor prognosis. Future studies should expand sample sizes and extend follow-up periods.

## 6 Conclusion

This study constructed and clinically validated an evidence-based, systematic, individualized, and multidisciplinary early postoperative home dietary management program addressing the common malnutrition risks in patients with preventive ileostomy. Preliminary application demonstrates that the program effectively improves short-term postoperative nutritional status (serum albumin, lymphocyte count, PG-SGA scores) and reduces HOS incidence. A positive trend was also observed in reducing peristomal moisture-associated skin damage (PMASD) incidence, though these differences did not reach statistical significance due to sample size and study design limitations. Future research with larger samples, more rigorous designs, and longer follow-up periods is needed to further validate the program's long-term effects, complication prevention efficacy, and impact on patient quality of life.

## Data Availability

The raw data supporting the conclusions of this article will be made available by the authors, without undue reservation.
